# Dupilumab improves clinical outcome assessments in children with eosinophilic esophagitis in a randomized phase 3 study

**DOI:** 10.1186/s12876-026-05017-8

**Published:** 2026-07-29

**Authors:** Mirna Chehade, Jonathan M. Spergel, Salvatore Oliva, Evan S. Dellon, Dhandapani Ashok, Marc E. Rothenberg, Ruiqi Liu, Margee Louisias, Allen Radin, Sarette T. Tilton, Eilish McCann

**Affiliations:** 1 Mount Sinai Center for Eosinophilic Disorders, Icahn School of Medicine at Mount Sinai, New York, NY USA; 2 Division of Allergy and Immunology, Children’s Hospital of Philadelphia, Department of Pediatrics, Perelman School of Medicine at University of Pennsylvania, Philadelphia, Pennsylvania USA; 3 Pediatric Digestive Endoscopy, Pediatric Gastroenterology and Liver Unit, Maternal and Child Health Department, University Hospital Umberto, Sapienza University of Rome, Rome, Italy; 4 University of North Carolina School of Medicine, Chapel Hill, North Carolina USA; 5 Division of Pediatric Gastroenterology and Hepatology, Department of Pediatrics, Children’s Hospital, London Health Sciences Centre, Western University, London, Ontario Canada; 6 Division of Allergy and Immunology, Department of Pediatrics, Cincinnati Children’s Hospital Medical Center, University of Cincinnati College of Medicine, Cincinnati, Ohio USA; 7 Regeneron Pharmaceuticals Inc., Tarrytown, New York USA; 8 Sanofi, Cambridge, Massachusetts USA

**Keywords:** Biologic therapy, Eosinophilic esophagitis, Patient-reported outcomes, Pediatrics, Quality of life

## Abstract

**Background:**

Eosinophilic esophagitis (EoE) is a chronic, progressive disease impacting patient quality of life (QoL). Assessment of pediatric EoE symptoms and their impact is challenging. This post hoc analysis of the phase 3 EoE KIDS trial (NCT04394351) evaluated a range of clinician-, caregiver-, and patient-reported outcomes (ClinROs/ObsROs/PROs) in pediatric patients aged 1–11 years with active EoE.

**Methods:**

EoE KIDS evaluated the efficacy and safety of dupilumab in a 16-week, placebo-controlled, double-blind period (Part A). ClinRO/ObsRO/PRO measures included the Pediatric EoE Sign/Symptom Questionnaire caregiver (PESQ-C) and patient (PESQ-P) versions, Pediatric EoE Symptom Score version 2.0 (PEESS v2.0-Caregiver version), Global Impression of Severity (GIS), Global Impression of Change (GIC), and Pediatric EoE Impact Scale (PEIS). EoE KIDS was not powered to detect significant differences in the main symptom measure, PESQ-C.

**Results:**

In Part A, 37 patients were assigned to higher-exposure dupilumab and 34 to placebo. The percentage reduction (improvement) from baseline to week 16 (least-squares mean [95% confidence interval]) in proportion of days with ≥1 EoE sign assessed by PESQ-C was numerically greater with dupilumab (-48.5% [-73.4, -23.6]) than placebo (-19.65% [-45.3, 6.0]). Changes in symptoms and QoL assessed by PEESS v2.0-Caregiver version, GIS, GIC, and PEIS instruments also generally favored dupilumab vs. placebo. High concordance was observed between individual signs of EoE (symptoms observed by caregivers) and corresponding patient-reported symptoms using PESQ-C and PESQ-P.

**Conclusions:**

Improvements with dupilumab vs. placebo were observed in most ClinROs and ObsROs, but these improvements were not consistent within certain PROs.

**Trial registration:**

ClinicalTrials.gov ID NCT04394351; Trial registered on May 7th, 2020.

**Graphical Abstract:**

**Figure:**
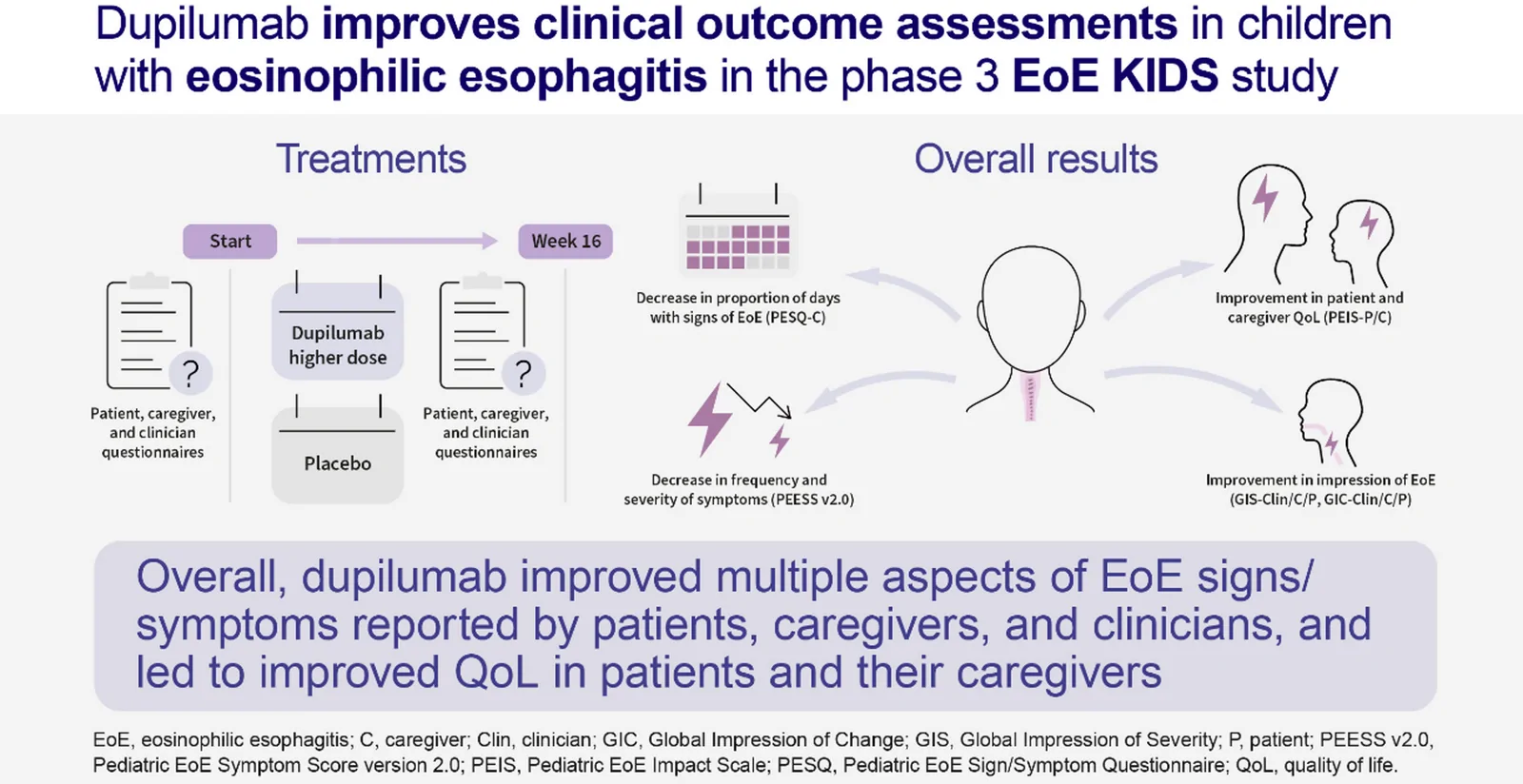

**Supplementary Information:**

The online version contains supplementary material available at https://doi.org/10.1186/s12876-026-05017-8.

## Background

Eosinophilic esophagitis (EoE), a chronic, progressive, type 2 inflammatory disease of the esophagus, has a substantial impact on patient quality of life (QoL) [1–4]. Reductions in QoL associated with EoE are also seen for caregivers, including social isolation and reduced ability to work [2, 5–9].

Adults and children with EoE have a similar esophageal histologic profile, and both may experience disassociation between histologic disease activity and symptoms [10, 11]. However, young children often present with more heterogeneous and non-specific symptoms than adults and adolescents, including abdominal pain, vomiting, food refusal, and heartburn [11–16]. Moreover, pediatric patients may struggle to articulate their symptoms due to limited verbal and cognitive development [12, 17–19]. Consequently, it is recommended that EoE symptoms are assessed by patients and their caregivers where possible, or by caregivers alone [11, 12, 20].

Several EoE-specific clinician-, caregiver-, and patient-reported outcome (ClinRO/ObsRO/PRO) measures have been developed to assess symptoms and QoL in children and adolescent patients up to 18 years of age, including the Pediatric EoE Symptom Score version 2.0 (PEESS v2.0)-Caregiver version and the Pediatric Quality of Life Inventory-EoE module version 3.0 (PedsQL-EoE) [21, 22]. Although existing measures have been validated for use in EoE studies, their accuracy in capturing child-experienced EoE symptoms remains uncertain, due to issues such as a long recall period (1 month for PedsQL-EoE and PEESS v2.0-Caregiver version) or requiring caregiver assessment of symptom severity [18, 23–26].

The Pediatric EoE Sign/Symptom Questionnaire (PESQ) was developed to address these issues and capture daily symptoms in children with EoE [27]. Patient (PESQ-P) and caregiver (PESQ-C) versions allow self-reporting in children where age-appropriate (≥8–<12 years), and caregiver assessment of signs (i.e., symptoms observed by the caregiver), to mitigate children’s limited capacity to report their symptom experience [28, 29].

Dupilumab, a fully human monoclonal antibody that blocks interleukin-4 and -13 signaling, is approved in the US, EU, and Canada for treatment of patients with EoE aged ≥1 year, weighing ≥15 kg [30–32]. In adult and adolescent patients with EoE, dupilumab 300 mg weekly demonstrated significant improvements in histologic, endoscopic, symptomatic, and molecular outcomes, with an acceptable safety profile in the phase 3 LIBERTY EoE TREET study (NCT03633617) [15, 33]. In the phase 3 EoE KIDS study (NCT04394351), weight-tiered dupilumab also demonstrated significant improvements to histologic, endoscopic, and transcriptional outcomes vs. placebo in patients with EoE aged 1–11 years, with a safety profile similar to that observed in adult and adolescent patients [34]. The EoE KIDS study was not powered to detect symptom differences.

Although most patients with EoE report that symptom and QoL improvements are the most important treatment outcomes, data on the impact of current therapies on these aspects are limited [35–38]. This post hoc analysis of EoE KIDS evaluates a range of ClinRO, ObsRO, and PRO measures to gain an overall understanding of the efficacy of dupilumab on signs/symptoms and QoL in pediatric patients aged 1–11 years with EoE [34].

## Methods

### Study design

The EoE KIDS study has been previously described [34]. Part A was a 16-week, randomized, double-blind, placebo-controlled treatment period. We report findings from Part A as clinical outcome assessment (COA) results may be impacted by patients’ positive expectations when receiving active treatment [39].

### Patients

Eligible patients were aged ≥1–<12 years with a confirmed diagnosis of active EoE (peak eosinophil count ≥15 eosinophils per high-power field (eos/hpf) in ≥2 of 3 esophageal regions: proximal, mid, or distal) that was unresponsive to ≥8 weeks of proton-pump inhibitor (PPI) therapy, with a clinical history of EoE-related symptoms in the month before screening. There were no inclusion criteria related to symptom severity. Written informed consent was obtained before enrollment from a parent or legal guardian, with patients providing written assent, if required.

### Interventions and procedures

Randomization was stratified by weight group at baseline (≥5 kg–<15 kg, ≥15 kg–<30 kg, or ≥30 kg–<60 kg). Patients were randomized 2:2:1:1 to weight-tiered higher- or lower-exposure subcutaneous dupilumab (equivalent to the dupilumab 300 mg weekly and dupilumab 300 mg once every 2 weeks doses in adults and adolescents, respectively) [33], or to 2 placebo groups, for 16 weeks. This analysis included patients treated with higher-exposure dupilumab (similar to the regimen approved by the U.S. Food and Drug Administration and the European Medicines Agency) or placebo.

### Outcomes and assessments

ClinROs, ObsROs, and PROs included in this analysis (Table 1) were percentage change from baseline in proportion of days with ≥1 EoE sign/symptom as measured by PESQ-C/P [27], PEESS v2.0-Caregiver version total and domain scores [21], Global Impression of Severity – Clinician/Caregiver/Patient (GIS-Clin/C/P), and absolute change from baseline in Pediatric EoE Impact Scale – Caregiver/Patient (PEIS-C/P). Notably, the PEIS-C instrument captures the impact of the patient’s EoE on caregiver QoL. Global Impression of Change – Clinician/Caregiver/Patient (GIC-Clin/C/P) was reported as proportion of patients in each response category.

**Table 1:** Sign/symptom and QoL outcome measures in the EoE KIDS study

Outcome measure	Main outcome	Outcome domains	Score range	Recall period
Patient symptoms/signs				
PESQ-C/P [27]	Proportion of days with ≥1 EoE sign/symptom	Stomach pain, heartburn, acid reflux, regurgitation, vomiting, food refusal, trouble swallowing food, and food stuck in throat^†^	0–1; higher scores indicate a greater proportion of days with ≥1 EoE sign/symptom	Daily
PEESS v2.0-Caregiver version [21]	Frequency and severity of EoE symptoms	There are 20 items across 4 domains (dysphagia, nausea/vomiting, GERD, and pain). Frequency and severity of symptoms: chest pain, heartburn, stomach ache, trouble swallowing, food stuck, drink to swallow, vomiting, nausea, regurgitation. Frequency only: eating less than others, eating longer than others	0–100; higher scores indicate greater symptom burden	Monthly
GIS-Clin/C/P^††^	Severity of EoE	Single-item measure of severity of EoE	4-point scale; 1 = mild, 4 = very severe	Weekly
GIC-Clin/C/P^‡^	Overall change from baseline in EoE	Single-item measure of overall change from baseline in EoE	7-point scale; 1 = very much improved, 7 = very much worse	Weekly
QoL of patients and of caregivers				
PEIS-P	Patient experience of their own QoL	How worried due to EoE, how sad due to EoE, how often embarrassed of EoE, how often difficulty sleeping, difficulty concentrating due to EoE, difficulty playing due to EoE	0–4; higher scores indicate poorer QoL	Weekly
PEIS-C	Caregiver experience of their own QoL	How worried due to EoE, how sad due to EoE, limited social activities, productivity limited, everyday activities limited, problems with relationship	0–4; higher scores indicate poorer QoL	Weekly

Caregiver- and clinician-reported questionnaires were completed for children of all ages. Patient self-assessment questionnaires were completed in children aged ≥8–<12 years

*Abbreviations:* *EoE *eosinophilic esophagitis, *GERD *gastrointestinal reflux disorder, *GIC-Clin/C/P *Global Impression of Change – Clinician/Caregiver/Patient, *GIS-Clin/C/P *Global Impression of Severity – Clinician/Caregiver/Patient, *PEESS v2.0 *Pediatric EoE Symptom Score version 2.0, *PEIS-C *Pediatric EoE Impact Scale – Caregiver, *PEIS-P *Pediatric EoE Impact Scale – Patient, *PESQ-C/P *Pediatric EoE Sign/Symptom Questionnaire caregiver/patient, *QoL *quality of life

^†^“Food stuck in throat” is a sub-question to “trouble swallowing food” and excluded from item-level analysis

^††^GIS-P has a 4-point scale; 1 = not bad, 4 = very bad

^‡^GIC-P has a 7-point scale; 1 = a lot better, 7 = a lot worse

Caregivers and patients were given training to complete the PESQ [39, 40]. The need to capture results independently was emphasized, and a single caregiver was encouraged to report signs in their child. The novel, validated PESQ-C tool defines caregiver-observed symptoms as signs; however, the other caregiver-observed measures used in this study describe caregiver-observed symptoms as symptoms, and the original language has been maintained for these measures [27, 41–43].

The signs/symptoms captured by PESQ-C and PESQ-P include stomach pain, heartburn, acid reflux, regurgitation, vomiting, food refusal, trouble swallowing food, and food stuck in throat (the latter is a sub-question dependent on “trouble swallowing food” and excluded from the analysis of individual signs/symptoms). The proportion of days with ≥1 EoE sign/symptom is reported using a score ranging from 0 to 1, with higher scores indicating a greater proportion of days with ≥1 EoE sign/symptom. Analyzed data were obtained from completed daily electronic diary entries in the 2 weeks preceding baseline and 2 weeks preceding the week 16 visit [27]. To perform the valid analysis of PESQ-P- and PESQ-C-based outcomes, a minimum of 8 electronic diary entries (including days when patients/caregivers did not experience/observe any EoE sign/symptom) is required for each 14-day period. If there are fewer than 8 electronic diary entries for the 14-day period, the PESQ-P/PESQ-C score is considered to be missing for that period.

Concordance between PESQ-C and PESQ-P was calculated using data from patients aged ≥8–<12 years and their caregivers, pooling all treatment groups. Concordance was defined as the proportion of days in which caregivers and patients both reported “Yes” or both reported “No” for the occurrence of individual signs/symptoms. Proportion of days with concordance was calculated as mean (standard deviation [SD]) on a scale of 0–1. Concordance was analyzed for each sign/symptom, based on all observed values.

The PEESS v2.0-Caregiver version is an EoE-specific ObsRO measure completed by caregivers that assesses the frequency and severity of EoE symptoms among pediatric patients; 20 items assess symptoms over 4 domains, comprising dysphagia, nausea/vomiting, gastrointestinal reflux disorder, and pain. The PEESS v2.0-Caregiver version has a 1-month recall period. The total PEESS v2.0-Caregiver version score ranges from 0 to 100; higher scores indicate greater symptom burden.

GIS-Clin, -C, and -P are single-item measures of the severity of EoE over the past week, intended to be completed independently by clinicians, caregivers, and pediatric patients with EoE aged ≥8–<12 years, respectively. GIS-C and GIS-Clin have a 4-point scale from 1 = mild to 4 = very severe. GIS-P has a 4-point scale from 1 = not bad to 4 = very bad (response option language was amended to be more easily understood by children).

GIC-Clin, -C, and -P are single-item measures of the overall change in EoE from baseline, intended to be completed independently by clinicians, caregivers, and pediatric patients with EoE aged ≥8–<12 years, respectively. GIC-C and GIC-Clin have a 7-point scale from 1 = very much improved to 7 = very much worse. GIC-P has a 7-point scale from 1 = a lot better to 7 = a lot worse.

PEIS-C is an ObsRO measure completed independently by caregivers of pediatric patients with EoE aged ≥1–<12 years, assessing the impact of the patient’s EoE on the QoL of the caregiver, including anxiety, social and professional activities, activities of daily living, and relationships, during the past week. PEIS-P is a patient-reported outcome measure completed independently by pediatric patients with EoE aged ≥8–<12 years, assessing the impact of EoE on the patient’s QoL during the past week on the same domains as listed for PEIS-C.

Endoscopy and imaging procedures were performed after all other efficacy and safety assessments were completed.

### Statistical analyses

Efficacy analyses were performed using the full analysis set. All *p*-values are nominal. An analysis of covariance model was used for continuous measures at week 16 using treatment groups, randomization stratification factor, and relevant baseline measurement as a covariate. For PESQ-C/P analyses, missing data were imputed using the worst observation carried forward method when missing due to rescue treatment, adverse events, or lack of efficacy; for other reasons, a multiple imputation approach was used. Data were obtained from completed daily electronic diary entries during the 2 weeks preceding baseline and the 2 weeks preceding the week 16 visit [27]. For all other COA measures reported, data were as observed.

## Results

### Patients

Recruitment for Part A began on September 1, 2020; the date of the last week 16 visit in Part A was April 28, 2022. In Part A, patients were randomized to receive dupilumab (*n* = 37) or placebo (*n* = 34) for 16 weeks. Patient demographics and baseline characteristics have been previously published and were generally comparable between treatment groups [34], except for a lower percentage of patients taking a PPI at randomization in the placebo group and an imbalance in the mean proportion of days with ≥1 EoE sign/symptom (described below). At baseline, mean age (SD) was 6.8 (3.1) years in the dupilumab group and 7.2 (3.0) years in the placebo group. Seventeen patients in the dupilumab group and 17 patients in the placebo group were aged ≥8–<12 years and eligible to complete patient self-assessments.

### Patient symptoms and signs

#### PESQ

At baseline, the proportion of days (mean [SD]) with ≥1 EoE sign as assessed by PESQ-C in the dupilumab group and placebo group was 0.46 (0.38) and 0.53 (0.36), respectively, and for PESQ-P was 0.42 (0.43) and 0.52 (0.34) [34]. Additionally, 14% of patients (dupilumab, *n* = 8; placebo, *n* = 2) had zero days with ≥1 EoE sign as measured by PESQ-C at baseline.

**Fig. 1:**
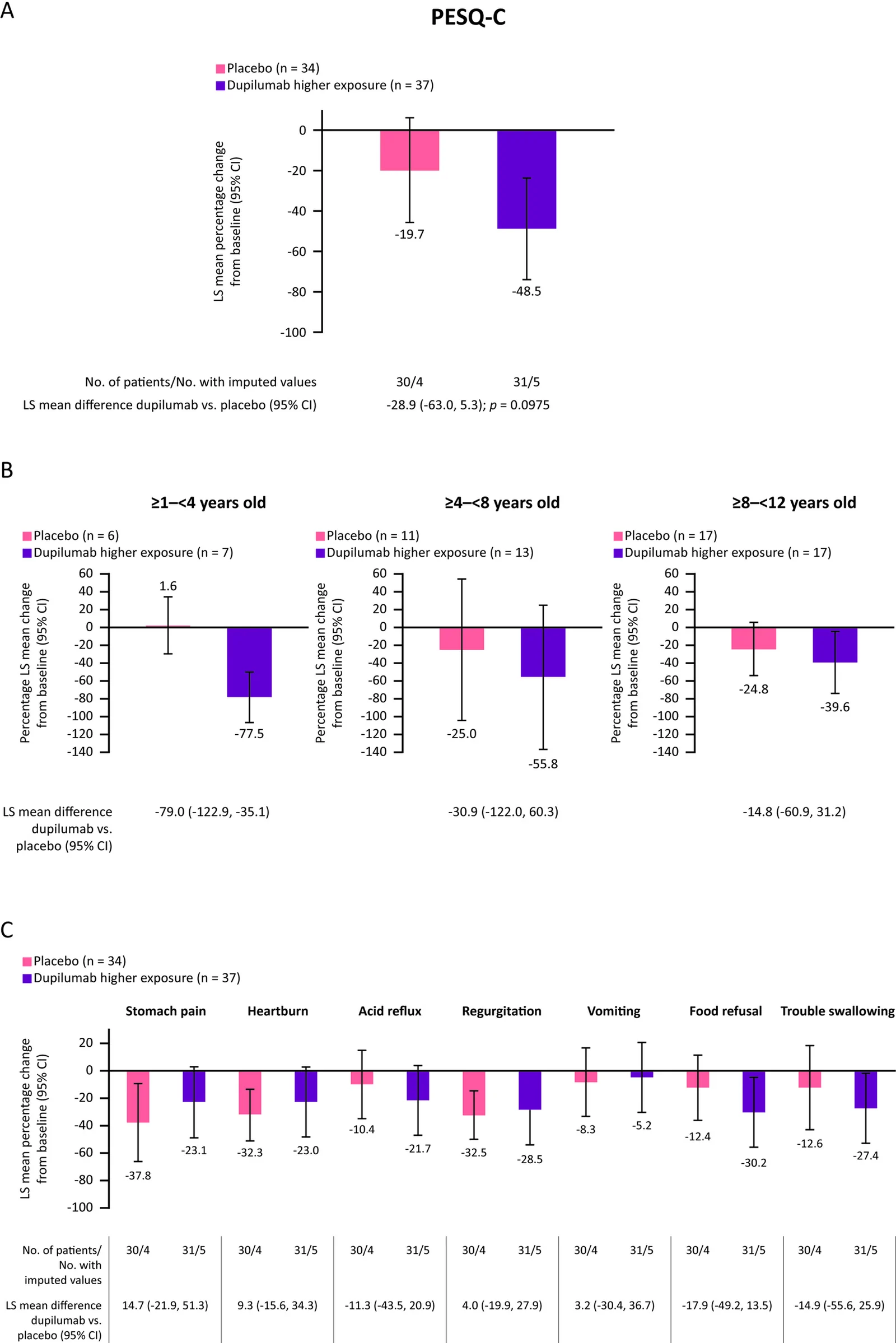
Change in caregiver-reported EoE signs measured by PESQ-C. Percentage change from baseline to week 16 in (**A**) proportion of days with 1 or more EoE sign/symptom as measured by caregivers (PESQ-C), p-value is nominal, (**B**) proportion of days with 1 or more EoE sign as measured by PESQ-C by patient age group, and (**C**) proportion of days with individual signs as measured by PESQ-C. Abbreviations: CI, confidence interval; EoE, eosinophilic esophagitis; LS, least-squares; PESQ-C, Pediatric EoE Sign/Symptom Questionnaire caregiver

Overall, patients showed a numeric reduction (improvement) from baseline in proportion of days with ≥1 EoE sign/symptom at week 16 [34]. The percentage change from baseline (least-squares [LS] mean [95% confidence interval (CI)]) at week 16 in proportion of days with ≥1 EoE sign as measured by PESQ-C was -48.5% (-73.4, -23.6) for dupilumab and -19.7% (-45.3, 6.0) for placebo (*p* = 0.1; Fig. 1A). Improvements were observed with dupilumab vs. placebo across 3 age subgroups, most notably in patients aged ≥1–<4 years (dupilumab, *n* = 7; placebo, *n* = 6) (Fig. 1B). Percentage change from baseline (LS mean [95% CI]) in proportion of days with individual EoE signs showed reductions in stomach pain (dupilumab: -23.1% [-48.8, 2.5]; placebo: -37.8% [-66.0, -9.6]), heartburn (-23.0% [-41.6, -4.4]; -32.3% [-51.1, -13.5]), acid reflux (-21.7% [-44.7, 1.4]; -10.4% [-35.3, 14.6]), regurgitation (-28.5% [-46.3, -10.7]; -32.5% [-50.4, -14.5]), vomiting (-5.2% [-30.1, 19.7]; -8.3% [-33.3, 16.6]), food refusal (-30.2% [-52.5, -7.9]; -12.4% [-36.3, 11.6]), and trouble swallowing (-27.4% [-57.6, 2.7]; -12.6% [-43.2, 18.0]) (Fig. 1C). For some signs, the average reduction at week 16 approached or equaled the average baseline value despite individual variability for each symptom reported, suggesting a high degree of sign resolution.

**Fig. 2:**
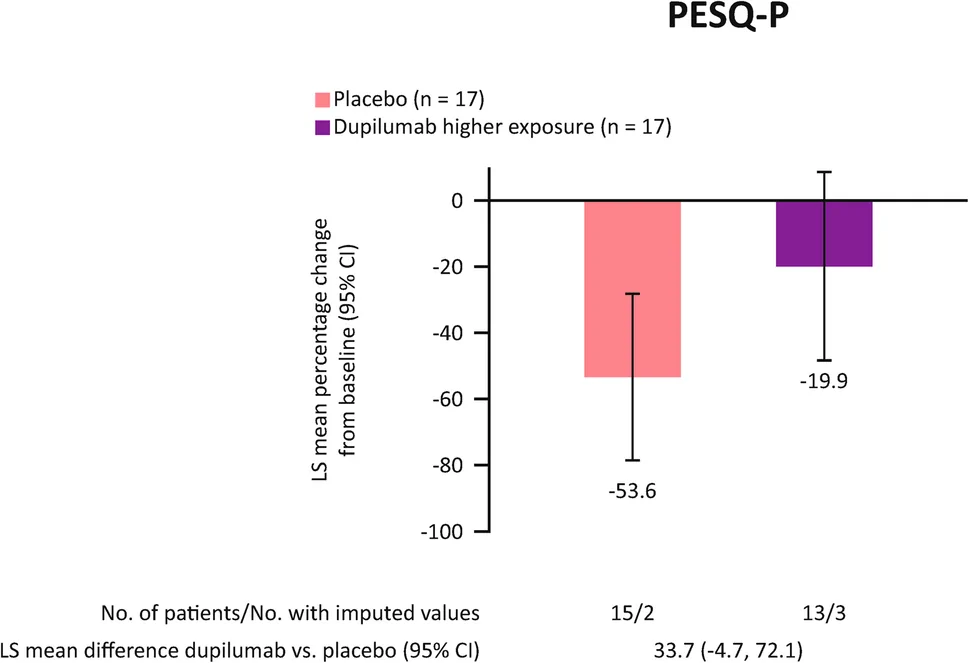
Change in patient-reported EoE symptoms measured by PESQ-P. Percentage change from baseline to week 16 in proportion of days with 1 or more EoE sign/symptom as measured by patients (PESQ-P). Abbreviations: CI, confidence interval; EoE, eosinophilic esophagitis; LS, least-squares; PESQ-P, Pediatric EoE Sign/Symptom Questionnaire patient

For PESQ-P (dupilumab, n = 17; placebo, n = 17), percentage change from baseline (LS mean [95% CI]) in proportion of days with ≥1 EoE symptom was -19.9% (-48.6, 8.8) and -53.6% (-78.4, -28.8) for dupilumab and placebo, respectively (Fig. 2).

**Fig. 3:**
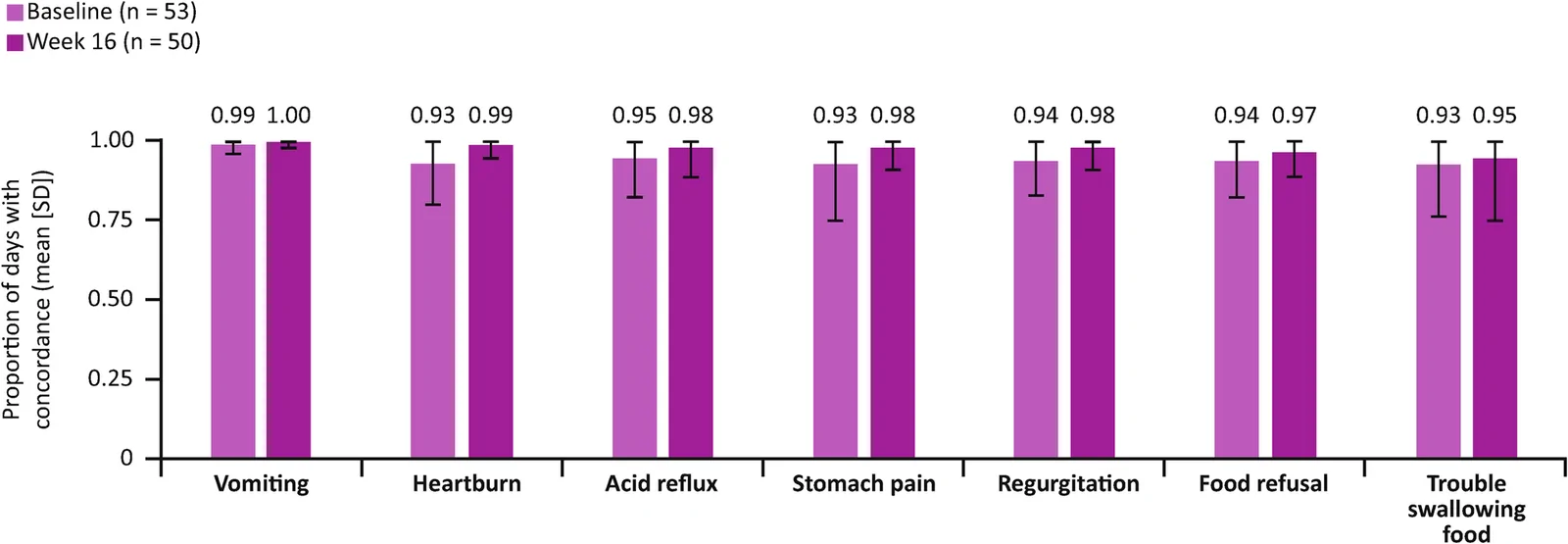
Concordance of caregiver- and patient-reported EoE symptoms. Proportion of days with concordance between PESQ-C and PESQ-P at baseline and week 16 for each sign/symptom^†^. Data are pooled for all treatment groups at baseline (*n* = 53) and week 16 (*n* = 50). Abbreviations: EoE, eosinophilic esophagitis; PESQ-C, Pediatric EoE Sign/Symptom Questionnaire caregiver; PESQ-P, Pediatric EoE Sign/Symptom Questionnaire patient; SD, standard deviation. Food stuck in throat is not reported, because this question was only asked if the patient experienced trouble swallowing. †Concordance is defined as both the caregiver and patient reporting “Yes/Yes” or “No/No” for each sign/symptom of EoE

There was a high proportion of days with concordance (mean [SD]) between PESQ-C and PESQ-P at baseline (≥0.93, *n* = 53), which was maintained through week 16 (≥0.95, *n* = 50). Median concordance at baseline and week 16 was 1.0 (complete concordance) for all symptoms (Fig. 3).

#### PEESS v2.0-Caregiver version

**Fig. 4:**
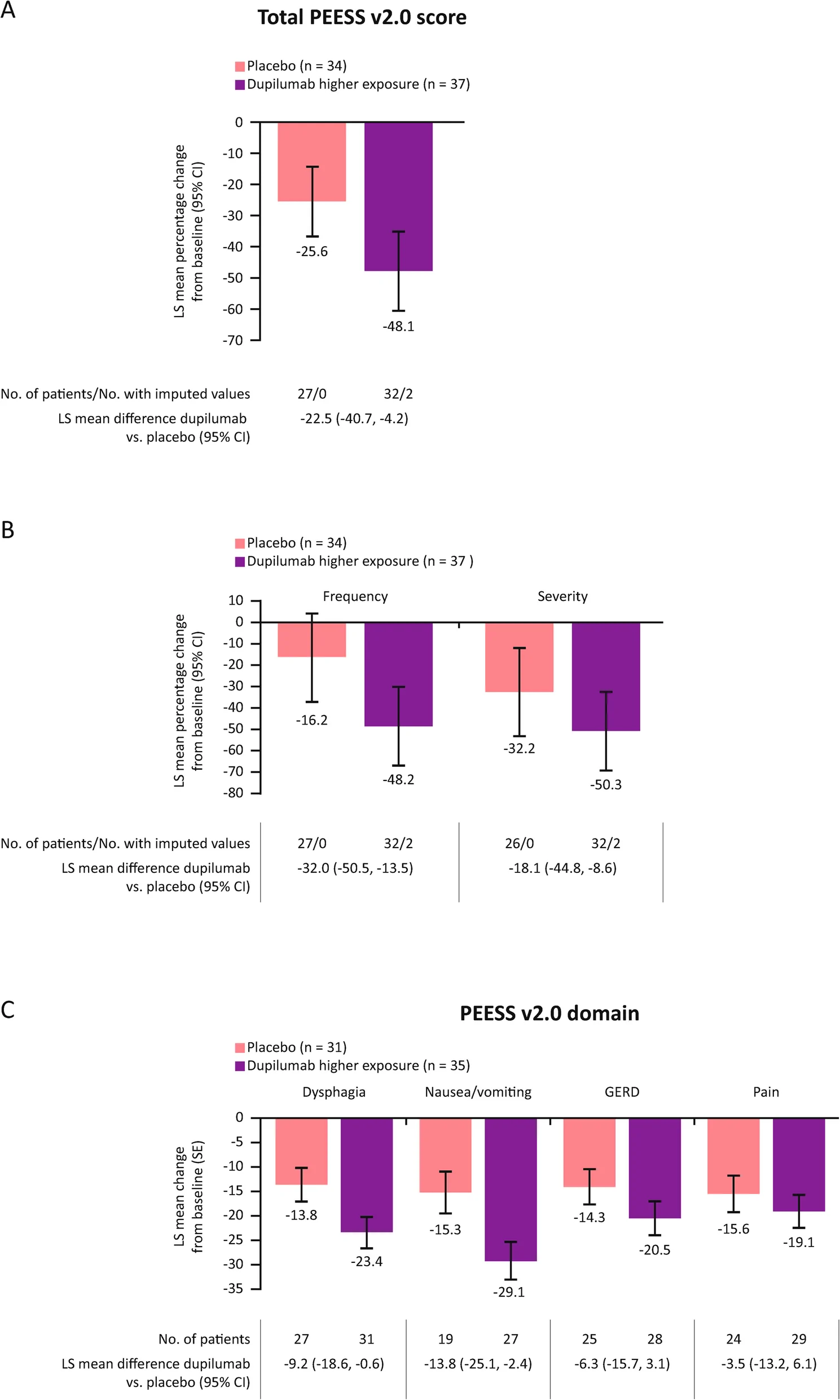
Change in EoE symptoms measured by PEESS v2.0-Cargiver version. Percentage change from baseline to week 16 in PEESS v2.0-Caregiver version (**A**) total score, (**B**) frequency and severity scores, and (**C**) change in domain scores. Total and domain scores are all scored 0–100. P-values are based on treatment difference (dupilumab group vs. placebo) of the LS mean percentage change or LS mean change using an analysis of covariance model with baseline measurement as a covariate, and the treatment and baseline weight group (≥5–<15 kg, ≥15–<30 kg, ≥30–<60 kg) strata as fixed factors. Abbreviations: CI, confidence interval; GERD, gastroesophageal reflux disorder; LS, least-squares; PEESS v2.0, Pediatric EoE Symptom Score version 2.0; SE, standard error

**Fig. 5:**
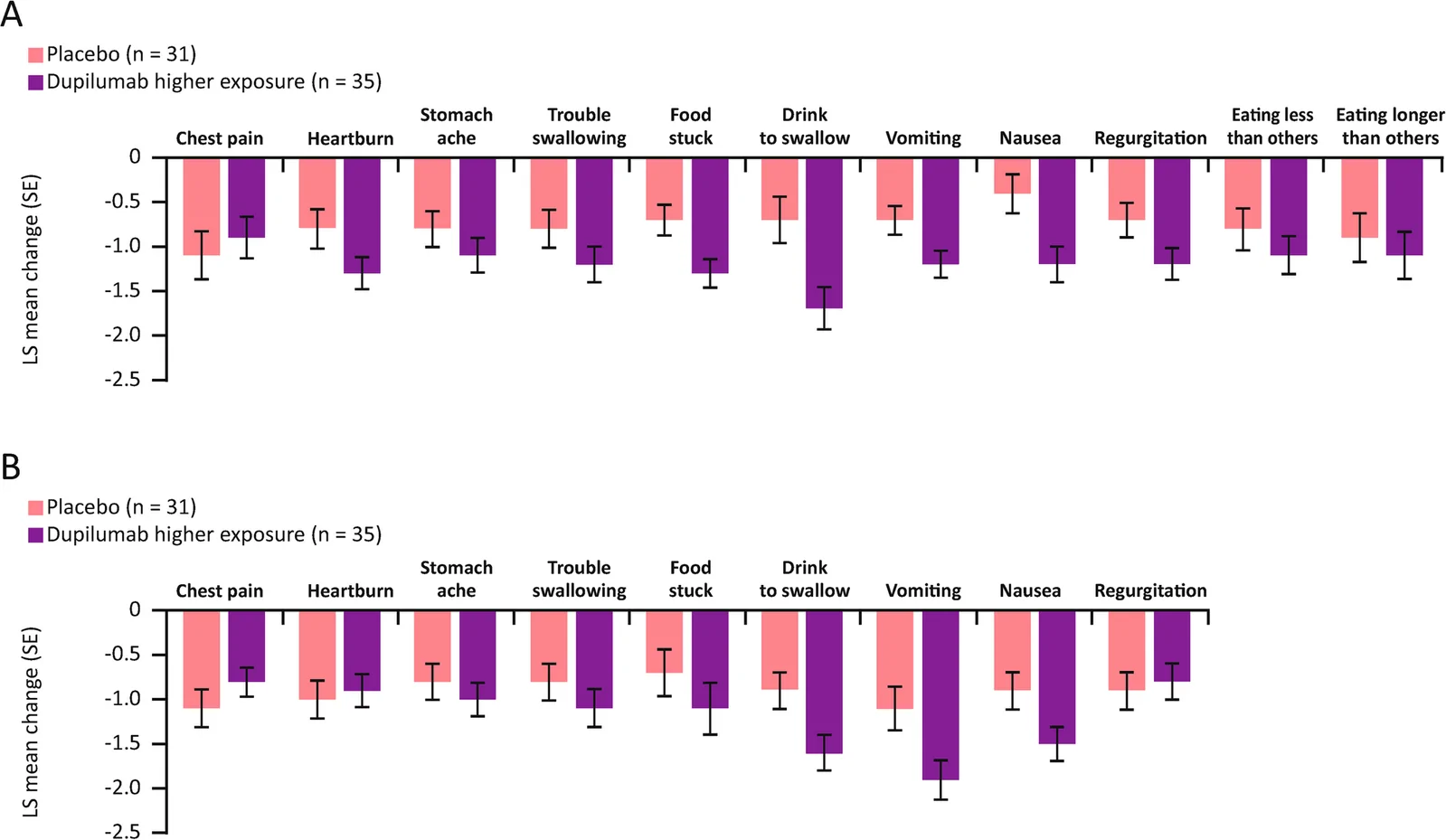
Change in frequency and severity of individual symptoms of EoE measured by PEESS v2.0-Cargiver version. Mean change from baseline in PEESS v2.0-Caregiver version individual item scores at week 16 by (**A**) frequency and (**B**) severity scores. Individual item scores are all scored 0–4. *P*-values are based on treatment difference (dupilumab groups vs. placebo) of the LS mean change using an analysis of covariance model with baseline measurement as a covariate, and the treatment and baseline weight group (≥5–<15 kg, ≥15–<30 kg, ≥30–<60 kg) strata as fixed factors.. Abbreviations: EoE, eosinophilic esophagitis; LS, least-squares; PEESS v2.0, Pediatric EoE Symptom Score version 2.0; SE, standard error

At baseline, mean (SD) PEESS v2.0-Caregiver version score was similar in dupilumab (38.0 [20.3]) and placebo groups (38.3 [17.8]) [34]. Patients showed greater mean percentage reduction (improvement) in PEESS v2.0-Caregiver version total score (95% CI) from baseline when treated with dupilumab (-48.1% [-60.9, -35.2]) vs. placebo (-25.6% [-39.8, -11.4]) at week 16 (Fig. 4A). Mean percentage change (95% CI) in symptom severity was − 50.3% (-68.9, -31.7) and − 32.2% (-53.0, -11.3) in the dupilumab and placebo groups, respectively (Fig. 4B). There was a greater mean percentage reduction (improvement) in symptom frequency with dupilumab compared with placebo (-48.2% [-61.2, -35.1] vs. -16.2% [-30.5, -1.8], respectively) (Fig. 4B). LS mean change from baseline (standard error [SE]) in PEESS v2.0-Caregiver version individual item frequency scores at week 16 showed reduced frequency in the “drink to swallow” (-1.7 [0.3] vs. -0.7 [0.3]), “nausea” (-1.2 [0.2] vs. -0.4 [0.2]), “food stuck” (-1.3 [0.2] vs. -0.7 [0.2]), and “vomiting” (-1.2 [0.2] vs. -0.7 [0.2]) items for dupilumab vs. placebo, respectively (Fig. 5A). At week 16, LS mean (SE) reductions (improvements) from baseline with dupilumab (vs. placebo) were seen in severity scores for “drink to swallow” (-1.6 [0.2] vs. -0.9 [0.2]) and “vomiting” (-1.9 [0.2] vs. -1.1 [0.3]) (Fig. 5B).

At week 16, greater numeric reduction was seen with dupilumab vs. placebo in dysphagia (-23.4 [3.3] vs. -13.8 [3.4]) and nausea/vomiting domain scores (-29.1 [3.9] vs. -15.3 [4.3]); LS mean (SE) numeric reductions from baseline were observed for dupilumab in gastroesophageal reflux disorder (-20.5 [3.4]) and pain (-19.1 [3.4]) domain scores (Fig. 4C).

#### Global assessments

**Fig. 6:**
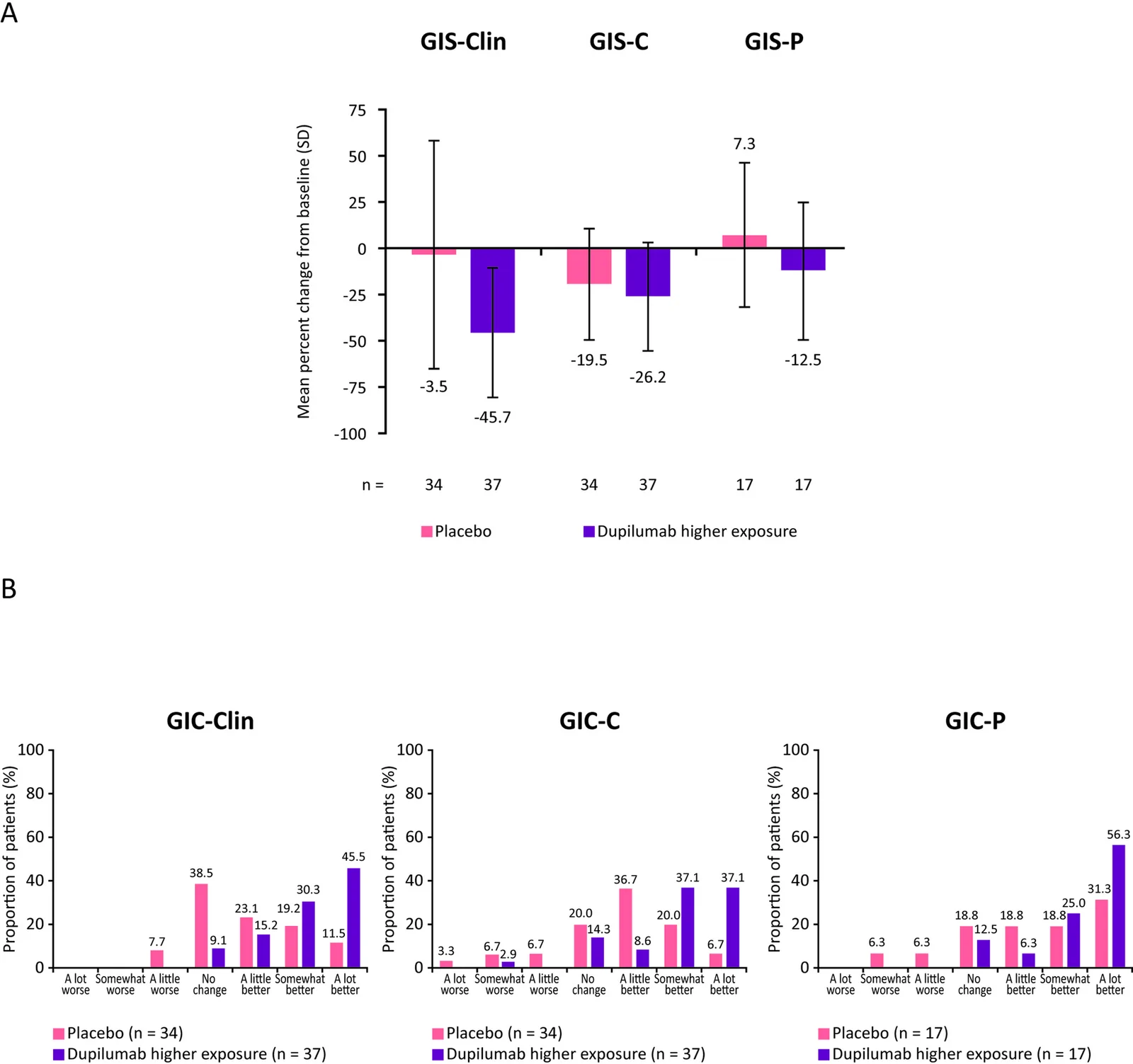
Change in impression of symptoms measured by GIS and GIC. **A** Percent change from baseline to week 16 in GIS-Clin, -C, and -P, and **B** proportion of scores at week 16 for GIC-Clin, -C, and -P. Abbreviations: GIC-Clin/C/P, Global Impression of Change-Clinician/Caregiver/Patient; GIS-Clin/C/P, Global Impression of Severity-Clinician/Caregiver/Patient; SD, standard deviation

At baseline, mean (SD) GIS-Clin/C/P scores were similar in dupilumab (2.4 [0.8], 1.9 [0.8], 1.8 [0.7], respectively) and placebo groups (2.0 [0.8], 1.8 [0.9], 1.6 [0.6], respectively). EoE disease severity, as measured by GIS-Clin/C/P, generally showed a mean (SD) percentage reduction (improvement) from baseline to week 16. Clinicians noted greater improvement in disease severity from baseline to week 16 for patients treated with dupilumab (-45.7% [35.2]) than placebo (-3.5% [61.8]) (Fig. 6A). Caregivers reported a similar improvement in disease severity from baseline in patients receiving dupilumab (-26.2% [29.5]) and placebo (-19.5% [29.9]) (Fig. 6A). Patients receiving dupilumab reported a -12.5% (37.3) numeric improvement in disease severity from baseline, whereas patients receiving placebo reported a 7.3% (39.0) numeric worsening (Fig. 6A).

Overall, responses to the 3 GIC questionnaires indicated that most patients experienced an improvement in disease status at week 16, especially those treated with dupilumab (Fig. 6B). Clinicians (GIC-Clin) reported 91.0% of patients treated with dupilumab as having an improvement, compared with 53.8% of those treated with placebo (Fig. 6B). Caregivers (GIC-C) reported an improvement in 82.8% of patients receiving dupilumab, compared with 63.3% receiving placebo (Fig. 6B). For patients using GIC-P, 87.5% reported an improvement with dupilumab compared with 68.8% with placebo (Fig. 6B), with 56.3% of those receiving dupilumab reporting their disease status as “a lot better”. No patients receiving dupilumab were reported to have worsened disease status per GIC-Clin and GIC-P. One caregiver of a patient receiving dupilumab scored the change in EoE status as “much worse” (2.9%; attributed by the caregiver to increased chest pain, stomach pain, and heartburn).

### Patient and caregiver QoL

#### PEIS

**Fig. 7:**
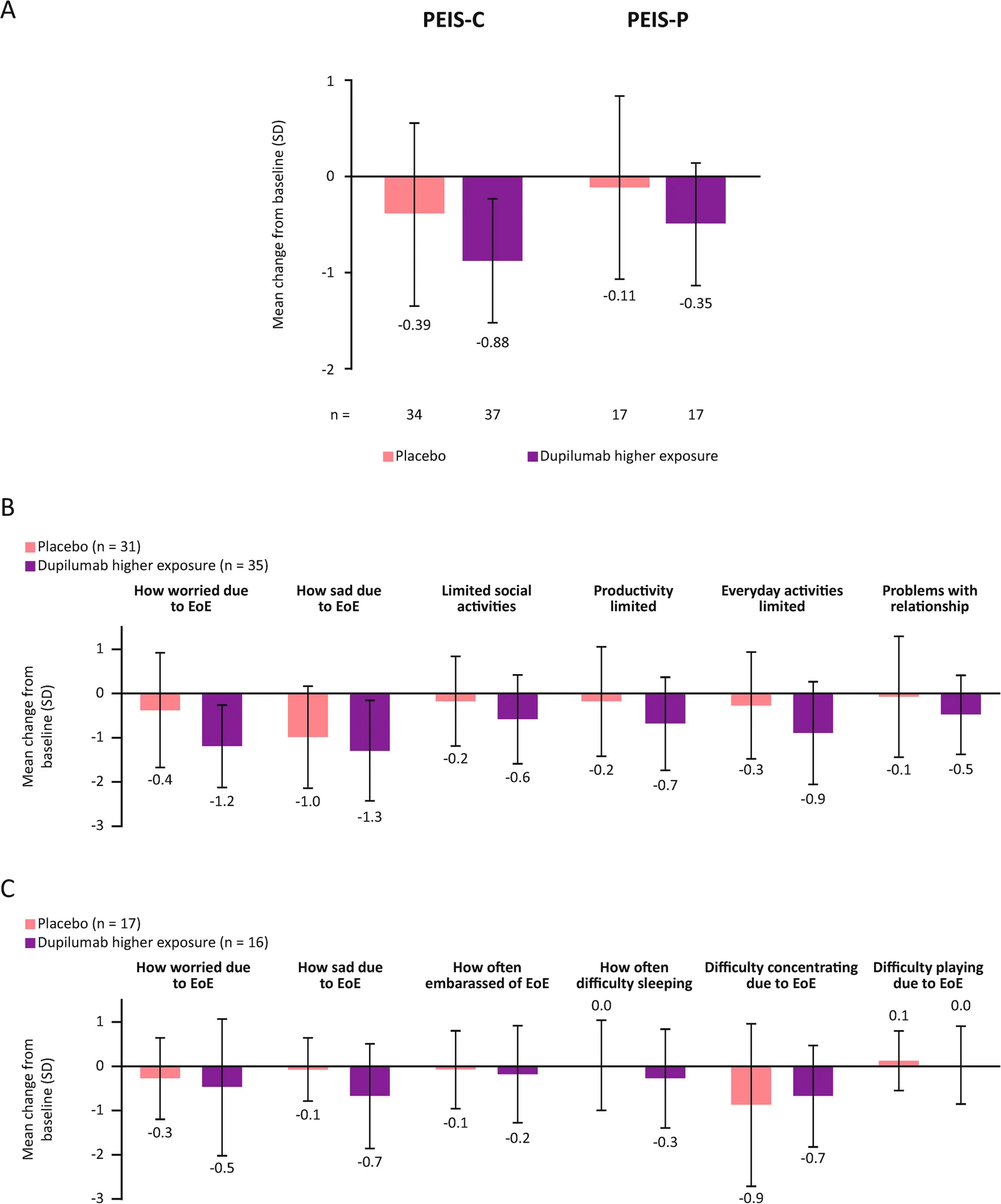
Change in impact of EoE on patient and caregiver quality of life measured by PEIS. Absolute change from baseline to week 16 in (**A**) PEIS-C and PEIS-P total scores, (**B**) PEIS-C individual item scores, and (**C**) PEIS-P individual item scores. Individual item scores are all scored 0–4. *P*-values are based on treatment difference (dupilumab groups vs. placebo) of the LS mean change using an analysis of covariance model with baseline measurement as a covariate, and the treatment and baseline weight group (≥5–<15 kg, ≥15–<30 kg, ≥30–<60 kg) strata as fixed factors. Abbreviations: EoE, eosinophilic esophagitis; LS, least-squares; PEIS-C, Pediatric EoE Impact Scale – Caregiver; PEIS-P, Pediatric EoE Impact Scale – Patient; SD, standard deviation

At baseline, mean (SD) patient-reported PEIS-P scores (dupilumab: 0.93 [0.71]; placebo: 0.54 [0.66]) were generally lower than caregiver-reported PEIS-C scores (dupilumab: 1.55 [0.80]; placebo: 1.34 [0.87]). Caregivers and patients in the dupilumab group reported reductions (improvements) in their QoL burden as measured by PEIS-C and PEIS-P from baseline to week 16 (Fig. 7A). Caregivers using PEIS-C reported greater mean (SD) absolute reduction in QoL burden from baseline with dupilumab (-0.88 [0.66]) than with placebo (-0.39 [0.96]) (Fig. 7A). Patients using PEIS-P reported greater absolute reduction in QoL burden with dupilumab (-0.35 [0.53]) than placebo (-0.11 [0.55]) (Fig. 7A). Caregivers also reported overall improvement from baseline to week 16 in PEIS individual item scores, with the dupilumab group showing ≥0.5-point reductions in all individual items (Fig. 7B). The greatest improvement was seen with dupilumab vs. placebo in the “how worried due to EoE” item (-1.2 [0.9] vs. -0.4 [1.3], respectively) (Fig. 7B). In general, patients using PEIS-P reported less improvement in QoL compared with caregivers using PEIS-C (Fig. 7C).

## Discussion

Assessment of pediatric EoE symptoms is challenging [11–13, 19, 44]. This analysis of children with active EoE aged 1–11 years is the first in pediatric studies to consider multiple COAs reported by patients, their caregivers, and their clinicians, allowing better insight into pediatric EoE symptoms and response to treatment. Greater improvements were generally observed with dupilumab vs. placebo after 16 weeks across the PESQ-C, PEESS v2.0-Caregiver version, GIS, and GIC instruments.

EoE KIDS was the first study to use PESQ, a tool specifically designed to address the challenges of assessing childhood EoE symptoms. Although the study could not be powered to detect significant differences in PESQ-C due to its novelty, a greater numeric percentage reduction from baseline in proportion of days with ≥1 EoE sign as measured by PESQ-C was seen with higher-exposure dupilumab than with placebo at week 16, with the same pattern observed across 3 age subgroups. In contrast, patients using PESQ-P reported greater improvement with placebo than dupilumab; however, the small sample size in this group and difficulty in assessing symptoms directly from young children limited interpretation of these data. Further, the placebo effect is commonly reported in patients with EoE, especially in children. A meta-analysis of 25 randomized clinical trials in adults and children identified a clinical response in 41% of patients who received placebo, with higher response rates associated with younger age [45]. Exit interviews conducted with patients and caregivers at the end of the placebo-controlled period confirmed the validity of the PESQ instruments (i.e., they measured relevant and present EoE signs/symptoms in pediatric patients). Quantitative analysis of improvement data collected during these interviews indicated that 95% of patients receiving dupilumab reported feeling better overall compared with 56% of patients receiving placebo [data not shown].

The high concordance between caregivers’ and patients’ assessments of individual EoE symptoms using PESQ-C and PESQ-P, respectively, at baseline and week 16 adds further evidence to the reliability of PESQ-C for use in EoE clinical trials and in settings where patients are untreated. These findings are consistent with studies showing that caregivers can accurately recognize and report patient-experienced symptoms in children aged ≥8–<12 years [8, 17, 46–48].

While patients and caregivers reported individual signs/symptoms with high concordance at baseline and week 16, they reported different trends in treatment effects for overall scores. This apparent contradiction may be explained by the small sample size in the PESQ-P analysis and the PESQ-C/P groups included in the concordance analysis, and differences in imputation and data aggregation between the efficacy and concordance analyses.

Similar to the PESQ-C, caregivers reported greater improvement in PEESS v2.0-Caregiver version total score from baseline with dupilumab than with placebo. Severity scores for “drink to swallow” and “vomiting” showed notable improvement using the PEESS v2.0-Caregiver version measure. These items have easily perceived visual cues for caregivers and it is possible that frequency of cues may subconsciously be used to assess severity (current guidance advises against assessing the severity of events such as vomiting) [27, 49]. Dysphagia-related individual symptoms were among the most improved items with dupilumab using both instruments (“drink to swallow” in PEESS v2.0-Caregiver version and “trouble swallowing” in PESQ-C). Dysphagia may instigate feeding difficulties that impact family mealtimes and meals at social events. Therefore, improvements in this sign/symptom with dupilumab may have contributed to improvements seen in individual items of PEIS in this study [44].

As pediatric care is centered around the family unit, the numerically greater improvement in QoL for patients treated with dupilumab, and their caregivers, compared with placebo—measured by PEIS-P and PEIS-C, respectively—is promising for effective EoE disease management at home. Notably, caregivers reported a greater improvement in QoL than patients, consistent with studies showing that even small reductions in caregiver burden can improve symptoms related to their burden, such as mood, coping, and self-efficacy [50].

Aspects of the study design limit interpretation of the data. EoE KIDS was only powered to show statistical significance in histologic remission, with a bridging strategy used to extrapolate other findings from adults to children [34]. EoE KIDS also had a relatively small study population, so it was not sufficiently powered to confirm differences between placebo and dupilumab in subgroups and individual symptoms. Additionally, a lack of inclusion criteria based on sign/symptom severity led to an imbalance in the proportion of days with ≥1 EoE sign/symptom reported by PESQ-C/P between dupilumab and placebo groups at baseline. This imbalance may not have had a large impact, because a quartile analysis of days with ≥1 EoE sign at baseline did not reveal appreciable differences between quartiles. However, part of this baseline imbalance was due to most patients with zero days with ≥1 EoE sign at baseline (14% of total patients) being assigned to the dupilumab group. Because these patients could not show improvement in the PESQ-C outcome, this may have limited the extent of potential improvements in the dupilumab group compared with the placebo group. As a result of these limitations, this study could not demonstrate consistent or statistically significant symptomatic improvements with dupilumab vs. placebo in patients with EoE aged 1–11 years.

## Conclusions

Relatively few recent clinical trials have assessed treatment efficacy in children with EoE [51–54]. The pediatric patients with EoE in the EoE KIDS study face a significant symptom and QoL burden, and capturing their symptoms presents unique challenges [11, 12, 20, 35–37]. Since post hoc analyses and study limitations precluded demonstration of statistically significant improvements, this analysis made use of multiple outcome measures assessing signs/symptoms and QoL. Most caregiver- and clinician-assessed versions of these outcome measures showed numeric improvements with dupilumab vs. placebo. However, the patient-reported versions of the outcomes did not consistently show improvements with dupilumab vs. placebo in the treatment of pediatric patients with EoE. Future research should consider larger patient populations to better understand the efficacy of dupilumab in providing symptomatic improvements for children with EoE.

## Supplementary Information

Supplementary Material 1.

## Data Availability

Qualified researchers may request access to study documents (including the clinical study report, study protocol with any amendments, blank case report form, statistical analysis plan) that support the methods and findings reported in this manuscript. Individual anonymized participant data will be considered for sharing once the indication has been approved by a regulatory body, if there is legal authority to share the data and there is not a reasonable likelihood of participant reidentification. Submit requests to https://vivli.org/.

## Abbreviations

EoE: Eosinophilic esophagitis
QoL: Quality of life
ClinRO: Clinician-reported outcome
ObsRO: Observer-reported outcome
PRO: Patient-reported outcome
PEESS v2.0: Pediatric EoE Symptom Score version 2.0
PedsQL-EoE: Pediatric Quality of Life Inventory-EoE module version 3.0
PESQ-P: Pediatric EoE Sign/Symptom Questionnaire patient
PESQ-C: Pediatric EoE Sign/Symptom Questionnaire caregiver
COA: Clinical outcome assessment
PPI: Proton-pump inhibitor
GIS: Global Impression of Severity
PEIS: Pediatric EoE Impact Scale
GIC: Global Impression of Change
SD: Standard deviation
LS: Least-squares
CI: Confidence interval
SE: Standard error
GERD: Gastroesophageal reflux disorder
