# Supplementary material for: Dupilumab improves clinical outcome assessments in children with eosinophilic esophagitis in a randomized phase 3 study
Source: BMC Gastroenterol. 2026 Jul 29;26:696. doi: 10.1186/s12876-026-05017-8 (PMC13700443; doi:10.1186/s12876-026-05017-8)
Supplement: Supplementary file 1 [file 12876_2026_5017_MOESM1_ESM.docx]

Dupilumab improves clinical outcome assessments in children with eosinophilic esophagitis in a randomized phase 3 study

**SUPPLEMENTARY MATERIAL**

**PLAIN LANGUAGE SUMMARY**

•	Why was this study done?

Eosinophilic esophagitis (EoE) is a long-term disease of the esophagus that can get worse over time and can negatively affect quality of life (QoL). In children, symptoms of the disease can be varied and may not be specific to EoE, such as abdominal pain, vomiting, food refusal, and heartburn. EoE can also make QoL worse for children’s caregivers. Dupilumab controls the underlying inflammation that drives EoE by blocking certain molecules involved in allergic reactions. Dupilumab is approved in the USA and EU for patients with EoE aged 1 year or older, weighing at least 15 kg. Symptoms and QoL can be difficult to assess in young children due to their inability to communicate what is happening to them. Therefore, in this study, researchers used multiple questionnaires aimed at children, caregivers, and clinicians to provide an overall assessment of symptoms and QoL in children with EoE, and QoL in their caregivers.

•	What did the researchers do?

The researchers looked at results from the EoE KIDS clinical trial, which included 102 children aged 1–11 years with EoE who received either a higher or lower dose of dupilumab, adjusted for their body weight, or placebo (inactive treatment) for 16 weeks. Questionnaires were completed by the children in the trial, their caregivers, and their clinicians at the start of the clinical trial (baseline) and 16 weeks after the start. The questionnaires assessing symptoms included the Pediatric EoE Sign/Symptom Questionnaire (PESQ; newly developed for this study), Pediatric EoE Symptom Score version 2.0 (PEESS v2.0), Global Impression of Severity (GIS), and Global Impression of Change. The impact of EoE on the QoL of patients and their caregivers was assessed using the Pediatric EoE Impact Scale (PEIS; newly developed for this study). Changes in the results of these assessments at week 16 of treatment were compared in patients who received higher-dose dupilumab or placebo.

•	What did the researchers find?

After 16 weeks of treatment, caregivers of children who received dupilumab reported fewer days with EoE symptoms using the PESQ than caregivers of patients on placebo. Caregivers also reported improvement in symptoms measured by PEESS v2.0, with notable improvements in symptoms related to difficulty swallowing and nausea. Additionally, clinicians reported improved EoE in the GIS. Improvements were also seen in the QoL of caregivers measured by PEIS, with notable reduction in the worry caused by their child’s EoE.

•	What do the findings mean?

As expected, there were difficulties assessing symptoms due to the relatively low patient numbers assessed. However, the evidence provided by multiple questionnaires suggests that dupilumab improves symptoms in children with EoE aged 1 year and older and weighing at least 15 kg, and improves QoL for their caregivers.
